# Relevance of gut microbiome research in food safety assessment

**DOI:** 10.1080/19490976.2024.2410476

**Published:** 2024-10-03

**Authors:** Manuel Garrido-Romero, Florencio Pazos, Elisa Sánchez-Martínez, Carlos Benito, José Ángel Gómez-Ruiz, Gonzalo Borrego-Yaniz, Cameron Bowes, Hermann Broll, Alberto Caminero, Eleonora Caro, Mónica Chagoyen, Marianne Chemaly, Antonio Fernández-Dumont, Haris Gisavi, Georgia Gkrintzali, Sangeeta Khare, Abelardo Margolles, Ana Márquez, Javier Martín, Caroline Merten, Antonia Montilla, Ana Muñoz-Labrador, Jorge Novoa, Konstantinos Paraskevopoulos, Cyrielle Payen, Helen Withers, Patricia Ruas-Madiedo, Lorena Ruiz, Yolanda Sanz, Rodrigo Jiménez-Saiz, F. Javier Moreno

**Affiliations:** a Department of Bioactivity and Food Analysis, Instituto de Investigación en Ciencias de la Alimentación (CIAL), CSIC-UAM, CEI (UAM+CSIC), Madrid, Spain; b Computational Systems Biology Group, National Centre for Biotechnology (CNB-CSIC), Madrid, Spain; c Department of Immunology, Instituto de Investigación Sanitaria Hospital Universitario de La Princesa (IIS-Princesa), Universidad Autónoma de Madrid (UAM), Madrid, Spain; d Instituto de Gestión de la Innovación y del Conocimiento, INGENIO (CSIC and U. Politécnica de Valencia), Valencia, Spain; e European Food Safety Authority (EFSA), Parma, Italy; f Institute of Parasitology and Biomedicine López-Neyra, CSIC, Granada, Spain; g Health Canada, Ottawa, ON, Canada; h Department of Food Safety, German Federal Institute for Risk Assessment (BfR), Berlin, Germany; i Department of Medicine, Farncombe Family Digestive Health Research Institute, McMaster University, Hamilton, ON, Canada; j Department of Medicine, McMaster Immunology Research Centre (MIRC), Schroeder Allergy and Immunology Research Institute (SAIRI), McMaster University, Hamilton, ON, Canada; k French Agency for Food, Environmental and Occupational Health and Safety, ANSES, Hygiene and Quality of Poultry, Pig Products Unit, Ploufragan, France; l National Center for Toxicological Research, US Food and Drug Administration, Jefferson, AR, USA; m Group of Functionality and Ecology of Beneficial Microorganisms (MicroHealth), Instituto de Productos Lácteos (IPLA-CSIC), Villaviciosa, Asturias, Spain; n Instituto de Investigación Sanitaria del Principado de Asturias (ISPA), Oviedo, Asturias, Spain; o Administration luxembourgeoise vétérinaire et alimentaire (ALVA), Strassen, Luxembourg; p Food Safety and Microbiology, Food Standards Australia New Zealand, Wellington, New Zealand; q Institute of Agrochemistry and Food Technology, Excellence Centre Severo Ochoa, Spanish National Research Council (IATA-CSIC), Valencia, Spain; r Department of Immunology and Oncology, National Centre for Biotechnology (CNB-CSIC), Madrid, Spain; s Faculty of Experimental Sciences, Universidad Francisco de Vitoria (UFV), Madrid, Spain

**Keywords:** Epithelial barrier, gut microbiota, metabolism, risk assessment, xenobiotic

## Abstract

The gut microbiome is indispensable for the host physiological functioning. Yet, the impact of non-nutritious dietary compounds on the human gut microbiota and the role of the gut microbes in their metabolism and potential adverse biological effects have been overlooked. Identifying potential hazards and benefits would contribute to protecting and harnessing the gut microbiome’s role in supporting human health. We discuss the evidence on the potential detrimental impact of certain food additives and microplastics on the gut microbiome and human health, with a focus on underlying mechanisms and causality. We provide recommendations for the incorporation of gut microbiome science in food risk assessment and identify the knowledge and tools needed to fill these gaps. The incorporation of gut microbiome endpoints to safety assessments, together with well-established toxicity and mutagenicity studies, might better inform the risk assessment of certain contaminants in food, and/or food additives.

Diet influences the composition and function of the human gut microbiota. Resident microbes and their activities impact many biological processes, such as metabolism, immunity, and the functioning of extra-intestinal organs (*e.g.*, brain, cardiovascular system, liver, and kidney).^1^ The human gut is densely colonized by microorganisms, especially by bacteria, belonging to more than 1,000 different species that together, with their collective genetic material and by-products, form the so-called gut microbiome. The gut microbiota is established at birth through contacts with the mother and the environment, starting a complex symbiotic interaction with the different host body’s systems. It can contribute to the host’s health status through multiple pathways, including protection against pathogens, drugs, industrial chemicals, environmental pollutants, and food components. The use of partially digestible and non-absorbable food additives and the presence of chemical contaminants in food has highly increased in recent years and, therefore, their impact on human health requires constant updates. Classical risk assessment approaches applied to foods paradoxically consider non- or even partially absorbable dietary compounds as inert upon excretion. This scenario overlooks the role that the gut microbiota and its metabolites play in bio-transformation and modulation of the biological activity of dietary compounds, as well as in their absorption into the bloodstream. Given the absence of a specific legal framework that considers microbiomes as an additional criterion for risk assessment, there is a lack of globally accepted methodologies and guidance to comprehensively address the impact of the gut microbiome changes on humans, animals, and the environment. This is in sharp contrast to the fact that many microbially processed compounds and/or derived metabolites are transported across the gut epithelium to the bloodstream.^2^ Indeed, the gut microbiome acts at the interphase between the dietary exposure and the host, and may play a significant role in determining the potential risks or benefits of non- or partially absorbable dietary compounds.^3^

Recent international initiatives have aimed to understand better the role of human microbiomes and their interactions with other microbial ecosystems in global health. The main goals of these initiatives were to define how the human microbiome affects health,^4–8^ and to identify dietary components that may harm (or be modulated by) the gut microbiome and the underlying molecular mechanisms. This article focuses on the interplay between dietary xenobiotics (*i.e.*, certain food additives and microplastics) and the gut microbiome with potential impact on human health. It also describes a series of research elements that could be part of safety studies to inform risk assessment and suggests research priorities to address knowledge gaps.

## Bibliographic methods used for gathering data

MEDLINE/PubMed (https://pubmed.ncbi.nlm.nih.gov/.) combined with records retrieved from Web of Science Core Collection (https://www.webofscience.com/wos/.), Scopus (https://www.scopus.com/) and ClinicalTrias.gov (https://clinicaltrials.gov/) were the literature databases selected to comprehensively cover the scientific evidence about the potential detrimental impact of dietary food additives and microplastics on the human gut microbiome. The general approach was based on the use of controlled terms complemented with queries using terms in titles, abstract, and author keywords, mainly limited to non-review papers published until April 2024. All retrieved articles were imported into the *ad hoc* web interface PMIDigest (https://github.com/JNovoaR/PMIDigest) which is a recently developed general-purpose tool for distilling large biomedicine-related bibliographic datasets.^9^

## The gut microbiome is a critical modulator of the catabolism of dietary components

Gut bacteria yield a plethora of metabolites (short-chain fatty acids (SCFAs), phenolic compounds, *etc*.) from dietary compounds, as well as micronutrients (*e.g*., vitamins). Some of those microbially produced metabolites are known to exert beneficial effects on immune maturation and function, epithelial barrier integrity and protection against infection.^10^ Gut microbes are also being increasingly recognized for their ability to communicate with the brain and modulate its function, through the production of neurotransmitters (*e.g*., histamine, gamma-aminobutyric acid, serotonin, and dopamine),^11^ and other bioactive metabolites (*e.g*., indoxyl sulfate, indole-3-acetic acid, indole-3-propionic acid, and 4-ethylphenol-sulfate), some of which are derived from (aromatic) amino acid catabolism.^12,13^ These metabolites can act locally in the gut, affecting the enteric nervous system or reaching distant organs affecting brain function.^14^ Altogether, microbial metabolism is key on intestinal homeostasis and human health.

**Figure 1.**
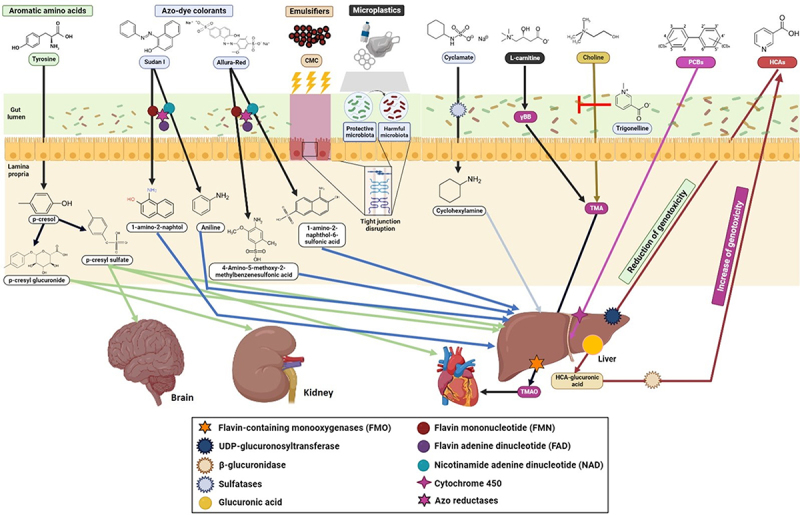
Overview of selected gut microbiome – dependent metabolic pathways of ingested xenobiotics with potential detrimental effects on different aspects of human physiology. The gut microbiome can modify the toxicokinetic and toxicodynamic of xenobiotics through different mechanisms by: i) direct metabolism through the reduction/increase of the compound activity (e.g., azo-dye colorants or PCBs) or re-activating inactive metabolites (e.g., HCAs) mediated by specific enzymes (e.g., azo-reductases, nitro- and nitrate-reductases, β-glucuronidases, β-glycosidases, sulfohydrolases, flavin-containing monooxygenases, β-lyases, organophosphorus hydrolases); ii) indirect influence of host metabolic and transport pathways in the liver and gut (e.g., alkaloids as trigonelline inhibiting gut microbial choline utilization and tma-forming ability); iii) inhibition (e.g., dietary emulsifiers) or promotion of bacterial growth affecting the composition and function of the gut microbiome; iv) up-regulation of genes associated with virulence factors. CMC: carboxymethylcellulose; γBB: γ-butyrobetaine; HCAs: heterocyclic amines; PCBs: polychlorinated biphenyls; TMA: trimethylamine; TMAO: trimethylamine N-oxide.

Conversely, the human gut microbiome may also undermine the host’s health by producing hazardous diet-derived metabolites. As an example, choline, betaine, or L-carnitine are bio-converted into trimethylamine (TMA) in the gut, which is further oxidized in the liver by flavin-containing monooxygenases resulting in trimethylamine N-oxide (TMAO)^15^ (Figure 1). The characterization of several microbial gene clusters, critical to this multi-step transformation pathway, linked microbial L-carnitine/choline catabolism of red meat with cardiovascular disease risk.^16,17^ Likewise, a range of xenobiotics used in agriculture, food, and pharmaceutical industries are known to interact with the gut microbiome, and could negatively influence human health through different mechanisms,^18–20^ as shown in Figure 1. Thus, alterations on intestinal microbial metabolism have been linked to different intestinal and metabolic disorders.

## The gut microbiome is impaired by certain dietary xenobiotics

**Figure 2.**
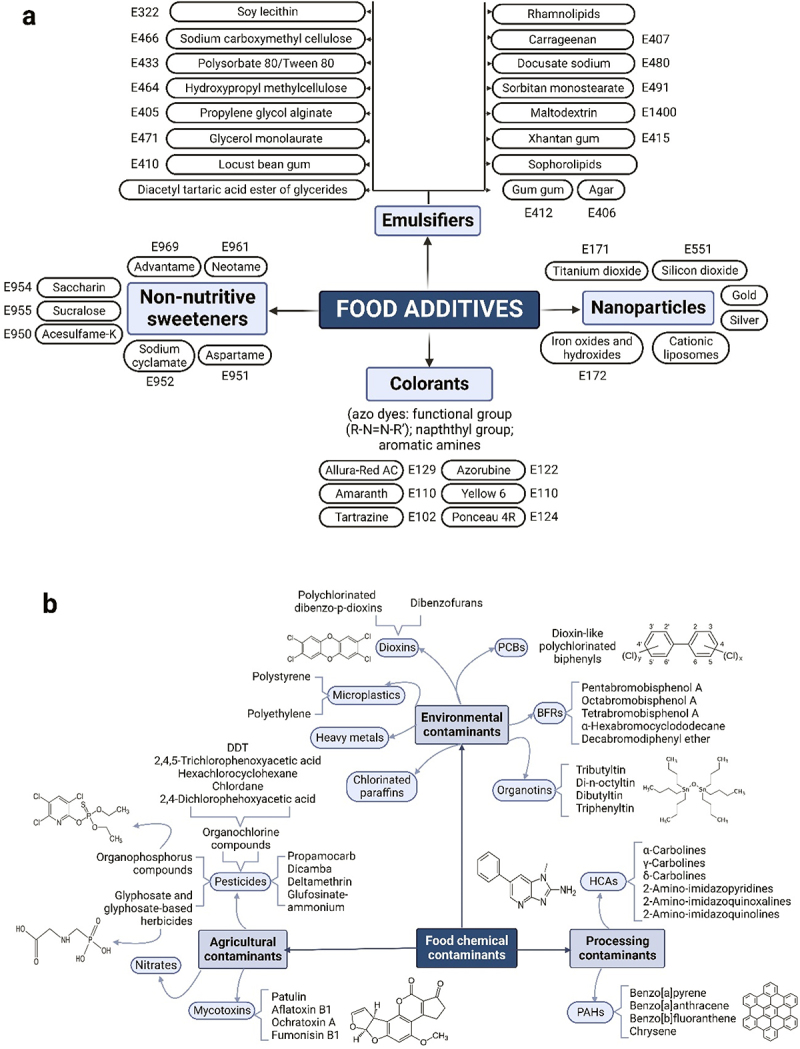
Reported (a) food additives and (b) chemical and/or microbial contaminants in food that could exert potentially detrimental effects on/by the human gut microbiome. Most of collected studies have been conducted *in vitro* or using murine models and their findings need to be treated with caution when translating them to humans due to the complexity of replicating host-microbiome interrelationship. These limitations need consideration when using data for human health risk assessments. PCBs: polychlorinated biphenyls; BFRs: brominated flame retardants; DDT: dichlorodiphenyltrichloroethane; HCAs: heterocyclic amines; PAHs: polycyclic aromatic hydrocarbons.

The use of food additives has increased recently in modern and Westernized diets linked to a higher consumption of processed foods,^21,22^ and microplastics are ubiquitous.^23^ The gut microbiome can be disturbed by certain dietary xenobiotics, such as additives (Figure 2(a)) and chemicals present in food (Figure 2(b)). According to current western dietary habits and lifestyle patterns, humans are highly exposed to xenobiotics from infancy and through the lifespan,^24^ which constitutes a global and emerging concern. The European Food Safety Authority (EFSA) as part of its risk assessment activities evaluates the safety of the food additives authorized in Europe. This risk assessment includes the dietary exposure to food additives; these exposure estimates are then compared to an adequate daily intake (ADI) derived from the hazard assessment. The ADI is an estimate of the amount of a substance in food or drinking water that can be consumed daily over a lifetime without presenting an appreciable risk to health. Here, we discuss the main interactions of the gut microbiome with synthetic colorants, non-nutritive sweeteners, emulsifiers, and microplastics, and their influence on host biological functions and health.

### Synthetic colorants

Artificial food colorants or dyes are substances made from petroleum (or crude oil) that enhance the color of processed foods. They are widely used in the food and pharmaceutical industries to increase the appeal and acceptability of their products. The use of synthetic colorants in dietary products has significantly increased over the past 50 years.^25^ These compounds can be metabolized in the gut releasing aromatic amines which are potentially carcinogenic and mutagenic^26,27^ as shown in Table 1.

**Table 1.** Gathered information linking selected synthetic colorants with changes/involvement of specific bacterial taxa, microbial metabolites and/or other end-products, and potential mechanism(s) influenced/mediated by the gut microbiome leading to adverse effects in the host.

Compound	Gut microbiota bacterial taxa associated to xenobiotic exposure	Microbial metabolites and pathway(s) on or mediated by the gut microbiome	Potential adverse effect(s) in the host	References
Allura Red AC (E129)	*In vivo* (mice)/*In vitro*: *Enterococcus faecalis; Bacteroides ovatus; Bacteroides fragilis; Bacteroides vulgatus; Odoribacter splanchnicus; Clostridium ramosum; Clostridium hylemonae; Faecalicoccus pleomorphus*	Enzyme bacterial-mediated transformations of azo-dyes colorants involving azo-reductases expressed by commensal bacteria into mainly sulfonated aromatic amines (*e.g.*, 1-amino-2-naphthol-6-sulfonate/cresidine-4-sulfonate in case of Allura Red; 1-amino-2-naphthol-disulfonates in case of amaranth or ponceau 4 R). These enzymes require NADH as electron donor and are mostly flavin mononucleotide (FMN) dependent	Allura Red promoted IBD-colitis in transgenic mice with dysregulated expression of interleukin (IL)-23 (Allura Red) or via colonic secretion of serotonin, microbial alterations and disruption of the epithelial barrier function	^28–33^
Yellow 6 (E110)	*In vivo* (mice)/*In vitro*: *Enterococcus faecalis; Enterococcus avium; Bacteroides ovatus; Escherichia coli*			
Tartrazine (E102)	*In vitro*: *Enterococcus faecalis; Escherichia coli; Lactobacillus; Enterobacter*			
Azorubine (E122)	*In vitro*: *Escherichia coli; Enterococcus*		*In vitro* assays indicated that the potential toxicity of azo-dye colorants might depend on the rate and extent at which the resident bacteria in the human gut microbiota reduces the azo linkage	
Amaranth (E123)	*In vitro*: *Enterococcus faecalis; Enterococcus avium; Bacillus cereum*			
Ponceau 4 R (E124)	*Escherichia coli; Enterococcus*			

The synthetic food colorant Allura Red (AR) is widely used in many countries and has been shown to enhance the susceptibility of mice to colitis via different mechanisms. First, azo-reductases produced by gut commensal bacteria (*e.g*., *Bacteroides ovatus* and *Enterococcus faecalis*) can release sulfonated aromatic amines leading to intestinal inflammation in IL-23 transgenic mice.^30^ A similar metabolic pathway has been described for Sudan I (Figure 1) and Yellow 6 colorants (Table 1). On the other hand, exposure to AR also enhances susceptibility to colitis via colonic secretion of serotonin, microbial alterations, and disruption of the epithelial barrier function. Indeed, AR exposure only induces mild colitis in naïve germ-free mice suggesting a pivotal role of the microbiota in mediating colorant-dependent inflammation.^34^ Of mention, some bacteria have the capacity to degrade dyes which may ultimately reduce toxicity in some individuals.^35^ However, the effects of these colorants on humans need to be further investigated. Furthermore, the most recent exposure data for AR refers to a refined exposure assessment conducted by EFSA in 2015.^36^ Among the different exposure scenarios assessed, in the refined brand-loyal assessment exposure scenario the mean exposure to AR ranged from 0.03 mg/kg body weight (bw) per day for the elderly to 1.4 mg/kg bw per day for toddlers. The high-level exposure ranged from 0.1 mg/kg bw per day for the elderly to 2.9 mg/kg bw per day for children.^36^ None of the dietary exposure scenarios assessed exceeded the ADI of 7 mg/kg bw per day established by the Joint FAO/WHO Expert Committee on Food Additives (JECFA) in 1980 and the EU Scientific Committee for Food (SCF) in 1984 and 1989.

### Non-nutritive sweeteners (NNS)

NNS are sugar substitutes that contain few to no calories but have a higher intensity of sweetness per gram. NNS have been broadly incorporated into foods such as beverages, frozen desserts, yogurts, snacks, and candies, as they are thought to contribute to combat obesity by providing a sweet taste but reducing the overall caloric intake. The most common NNS are saccharin, aspartame, sucralose, and cyclamate. There is controversy over the safety of consuming NNS. For example, a study showed that consumption of commonly used NNS drives the development of glucose intolerance through induction of compositional and functional alterations to the intestinal microbiota, with saccharin being the most deleterious^37^ (Table 2). Pathways overrepresented in saccharin-consuming mice include those involved in enhanced energy harvest and glycan degradation. Glycans are fermented to SCFAs, among others. Although SCFAs are linked to beneficial immune and metabolic effects, these metabolites have been previously associated to obesity. An increase on glycosaminoglycan sulfatases and glycoside hydrolases degradation pathway gene expression was also observed in fecal samples of saccharin-consuming mice. Sulfohydrolases may catalyze the transformation of cyclamate into cyclohexylamine,^41^ a metabolite responsible for the potential carcinogenic effect of cyclamate (Figure 1).

**Table 2.** Gathered information linking selected non-nutritive sweeteners with changes/involvement of specific bacterial taxa, microbial metabolites and/or other end-products, and potential mechanism(s) influenced/mediated by the gut microbiome leading to adverse effects in the host.

Compound	Gut microbiota bacterial taxa associated to xenobiotic exposure	Microbial metabolites and pathway(s) on or mediated by the gut microbiome	Potential adverse effect(s) in the host	Refs
Saccharin (E954)	*In vivo* (mice): an increase in relative abundance belonged to the *Bacteroides* genus and *Clostridiales* order *In vitro*: an increase of the *Bacteroidetes* phylum and reduction in *Firmicutes*	Microbial pathways overrepresented in saccharin-consuming mice include a strong increase in glycan degradation pathways, in which glycans are fermented to form various compounds including short chain fatty acids (SCFAs) like propionate and acetate. These pathways mark enhanced energy harvest and their enrichment was previously associated with obesity in mice and humans, with SCFA possibly serving as precursors and/or signaling molecules for de *novo* glucose and lipid synthesis by the host	Glucose intolerance obesity in mice and humans	^38–40^
	*In vivo* (rats): a decrease in *Akkermansia muciniphila* and an increase in *Firmicutes*	The reduction of *Akkermansia muciniphila* could be the result of a change in the mucin profile, fucosylated milk oligosaccharides and/or gut pH due to changes in metabolites, microbe abundance or inflammation status following pre- and post-natal maternal exposure to non-nutritive sweeteners	Metabolic disease and obesity in rats	
Sucralose (E955)	*In vivo* (mice): alterations in the families *Turicibacteraceae, Lachnospiraceae, Ruminococcaceae, Verrucomicrobiaceae, Staphylococcaceae, Streptococcaceae, Dehalobacteriaceae, Lachnospiraceae* and unclassified members in families *Clostridiaceae, Christensenellaceae, Peptostreptococcaceae, Erysipelotrichaceae* and in the order *Bacillales*	An increase of pro-inflammatory mediators, lipopolysaccharide (LPS), flagella, fimbriae, bacterial toxins, and a decrease of anti-inflammatory metabolites	Liver inflammation in mice which is confirmed by elevated gene expression of pro-inflammatory markers in the liver, such as matrix metalloproteinase 2 (MMP-2) and inducible nitric-oxide synthase (iNOS)	

In a randomized-controlled trial (RCT) on the effects of NNS in 120 healthy individuals, saccharin and sucralose consumption significantly impacted glucose tolerance, unlike aspartame and stevia. Microbiome changes were highly correlated with the alterations observed in individuals´ glycemic responses. Thus, the glycemic responses from conventionalized gnotobiotic mice with microbiomes from multiple top and bottom responders of each of the NNS-supplemented groups were largely reflecting those of their respective human donors. These findings suggest that gut microbiome alterations in humans following consumption of certain NNS could lead to metabolic person-dependent alterations.^40^

For saccharin, no recent exposure data are available from EFSA, although it seems there is an on-going risk assessment as indicated on their website.^a^ The most recent data on dietary exposure to saccharin is reported by Tennant^42^ In this study, dietary exposure to non-nutritive sweeteners consumed as tabletop sweeteners was assessed. Some reliable exposure estimates were produced for saccharin using the consumption data for tabletop sweeteners in powder from the EFSA Comprehensive European Food Consumption Database. The maximum mean and 95th percentile exposure across the European population were identified in the elderly and very elderly population (>64 years), with estimates of 0.16 mg/kg bw per day and 1.20 mg/kg bw per day, respectively. These estimates are well below the current ADI of 5 mg/kg bw per day set by JECFA in 1993 and the SCF in 1995.

### Emulsifiers

Emulsifiers are detergent-like food additives used to mix two immiscible substances, so they play a crucial role in the food and beverage industry. A RCT showed an association between carrageenan consumption and relapse in patients with ulcerative colitis in remission. An increase in the pro-inflammatory marker IL-6 and fecal calprotectin was also associated with carrageenan intake.^43^ It was hypothesized that this detrimental effect might be associated to carrageenan interaction with the intestinal microbiota, as well as to the direct activation of inflammatory pathways in the colonic epithelial cells. A recent study showed that carrageenan also impacts bacteria-derived SCFAs that could be the cause of intestinal inflammation^44^ (Table 3). Carrageenan-mediated inflammation is also associated with its unique chemical structure, which is based on a D-galactose (Gal) backbone alternating α-1,3 to β-1,4 linkages sulfated at up to 40%.^63^ Interestingly, α-1,3-galactosidic bonds have been associated with human rejection of vascularized organ transplants from pigs mediated by Gal-specific human IgM and IgG.^64,65^ Moreover, the disaccharide galactose-α-1,3-galactose is responsible for the α-gal syndrome, an allergic syndrome that typically presents as a delayed hypersensitivity due to the ingestion of red meat.^66,67^

**Table 3.** Gathered information linking selected dietary emulsifiers with changes/involvement of specific bacterial taxa, microbial metabolites and/or other end-products, and potential mechanism(s) influenced/mediated by the gut microbiome leading to adverse effects in the host.

Compound	Gut microbiota bacterial taxa associated to xenobiotic exposure	Microbial metabolites and pathway(s) on or mediated by the gut microbiome	Potential adverse effect(s) in the host	Refs
Sodium carboxymethyl cellulose (CMC) (E466) Polysorbate 80 (P80) (E433)	*In vivo* (mice): CMC male mice treated exhibited higher abundance of the genus *Dorea* CMC female mice showed increases in *Anaeroplasma* *In vivo* (mice): P80 male mice treated increased the abundance of the genera *Bacteroides*, *Burkholderia*, *Clostridium*, and *Veillonella* P80 female mice treated increased the relative abundance of the *Proteobacteria* phylum and of *Clostridium* and *Burkholderia* genus	Change in expression of neuropeptides (CMC and P80)	Sex-specific alterations of the microbiota may have led to sex-specific changes in behavior, altered anxiety-like behaviors in males and reduced social behavior in females (CMC and P80)	^44–62^
	No gut microbiota bacterial taxa associated	Microbiota encroachment into the mucus; including an increase of bacteria that produce proinflammatory flagellin and lipopolysaccharide (LPS) (CMC and P80)	Epidemiologic evidence and animal studies point out that CMC and P80 could play a role in contributing to the increased prevalence of diseases associated with chronic inflammation and metabolic disorders	
Polysorbate 80 (P80) (E433) Polysorbate 20 (E432)	*In vitro: Escherichia coli*	Mucosal microstructure and particle dispersion. It also increases the motility of *Escherichia coli* and its ability to translocate across microfold epithelial cells, through which the gut epithelium was invaded by intestinal microbiota	Cell death at concentrations between 1% and 0.1%. Even at concentrations lower than 0.1%, P80 and P20 induced a proinflammatory response suggesting a detrimental effect on gastrointestinal health	
Carrageenans (E407)	*In vitro:* Many species from the genus *Bacteroides* contain enzymes like carrageenanases, able to break carrageenans into oligosaccharides A carrageenan-degrading species from *Bacteroides*, *Bacteroides xylanisolvens* has been identified as a potential IBD biomarker	Carrageenans with α − 1→3 and β − 1→4 glycosidic linkages can be hydrolyzed by carrageenanases and α − 1→(3,6)-galactosidase, respectively. Interestingly, the hydrolysis of carrageenans by κ- or ι-carrageenanases promoted the expression of IL8 and BCL10 in human colonic epithelial cells.	Data collected by *in vitro* and animal studies suggest that the accumulation of this poligeenan can impair gut permeability and increase the risk of gut inflammatory incidence and even colon cancer.	
	No gut microbiota bacterial taxa associated	Carrageenans inhibit gastric proteolysis. The inadequately digested proteins are fermented by gut microbes in the colon, producing toxic metabolites, such as hydrogen sulfide, indole, and ammonia.	This tends to increase the risk of colorectal disorders.	
	No gut microbiota bacterial taxa associated	An increase in total sulfated glycosaminoglycans (GAGs) by decreasing the activity of various sulfatases. The decrease of heparin sulfamidase activity increases heparan sulfate, activating the Wnt/beta-catenin which plays a crucial role in intestinal polyp formation after injury. *In vitro* The levels of short-chain fatty acids (SCFAs), particularly butyrate, were significantly decreased in the colon or cecum. *In vivo* Caco2 cells have affected cytosolic plague proteins, such as zonula occludens proteins. Zo1 mediates the interaction between transmembrane proteins and the apical cytoskeleton in tight junctions, which hold keys to pathogen invasion. *In vitro*	Problems in polyp formation after injury. Alterations in colon or cecum. Improper pathogen invasion.	
Glycerol Monolaurate (E471)	***In** vivo* (mice): Decreased abundance of *Akkermansia muciniphila* and *Lupinus luteus* and increased abundance of *Escherichia coli* and *Bacteroides acidifaciens*	Significant change in the β-diversity and composition of gut microbiota and upregulation of the circulating levels of serum LPS, interleukin (IL)-1β, IL-6, and tumor necrosis factor (TNF)-α. Increased of several predicated metabolism pathways involved in carbohydrate, amino acid, and lipid metabolism	Promotion of metabolic syndrome, gut microbiota dysbiosis, and systemic low-grade inflammation in low-fat diet fed mice	

**Figure 3.**
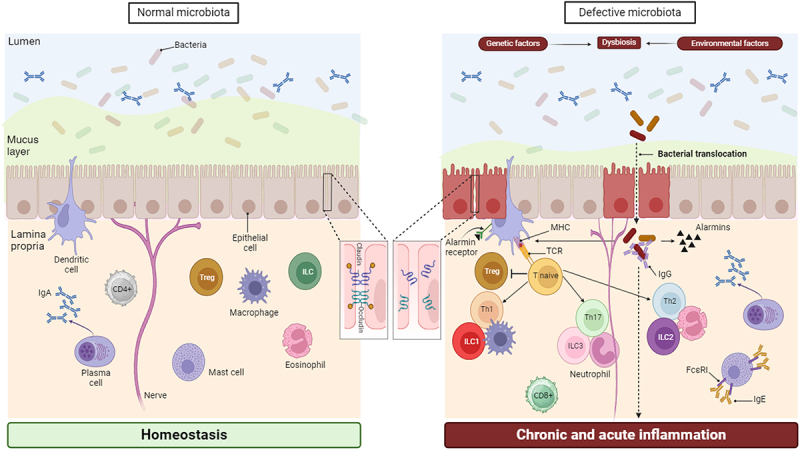
Key molecular events underlying host-gut microbiome interplays following exposure to potentially harmful dietary xenobiotics. metabolism, inflammation, immune responses, and intestinal barrier integrity are all crucially influenced by the gut microbiome. Evidence based on a limited number of research studies shows that exposure to certain dietary xenobiotics may alter gut homeostasis (left side) in different ways. These include affecting gut microbial composition, reducing the mucus layer, causing rupture of tight junctions, epithelial damage, and release of alarmins, increasing intestinal permeability, favoring translocation of commensal bacteria into the lamina propria, activation of dendritic cells and sensory neurons, etc. (right side). These processes are typical of altered host–microbiota interactions in chronic inflammatory diseases, such as IBD, and others (e.g., metabolic dysregulation, autoimmune diseases, hypersensitivity reactions) as compared to normal gut microbiota maintaining homeostasis (left side). All of them represent threats to gut homeostasis which dampen T-regulatory responses and lead to different types of innate (eosinophils, neutrophils, mast cells, innate lymphoid cells (ILC)), and adaptive inflammation (Th1, Th2, and Th17) including IgA and IgG response against commensal bacterial, and ultimately to disease manifestations.

Other emulsifiers such as carboxymethylcellulose (CMC) and polysorbate (P) 80 have been shown to induce low-grade inflammation and metabolic syndrome in wild-type mice, and colitis in predisposed mice. Emulsifier-induced metabolic syndrome was associated with microbiota alterations, bacterial encroachment, and increased pro-inflammatory response (Table 3). Bacterial invasion was marked by a significant thinning of the inner mucus layer due to the accelerated breakdown of mucus, whilst increased intestinal permeability is correlated with pro-inflammatory markers^57^ (Figure 3).

Furthermore, a RCT revealed that individuals who consumed CMC had increased postprandial abdominal discomfort and disturbed gut microbiota composition in a way that reduced its diversity. CMC-fed subjects experienced fecal metabolome changes, such as decreases in SCFAs and free amino acids. Two CMC-consuming subjects also showed an increased microbiota translocation into the inner mucus layer, a characteristic of altered host–microbiota interactions in chronic inflammation, as well as other alterations in microbiota composition^58^ (Table 3). In addition, human microbiota determine individualized inflammatory response to CMC.^68^ Recently, P20 and P80 have shown dose-dependent cytotoxicity and impaired epithelial barrier function and integrity on human gut epithelial cells and intestinal organoids.^61^ Biological processes including inflammation and oxidative stress, among others, were upregulated at the cellular level in response to both emulsifiers, thus creating a local environment prone to allergic sensitization or other inflammatory diseases.^69^ Overall, growing evidence suggest that certain emulsifiers could have deleterious effects in humans.

Finally, exposure data in the European population are available from EFSA on the combined of exposure of modified and unmodified celluloses (E 460–466, E 468, and E 469) that includes sodium CMC (E 466).^70^ As mentioned, CMC is one of the food additives reported to alter the gut microbiota together with other emulsifiers. From the refined estimated exposure scenario, in the brand-loyal scenario, mean exposure to cellulose (E 460–466, E 468, and E 469) from their use as food additives ranged from 5 mg/kg bw per day in infants to 190 mg/kg bw per day in toddlers. The high exposure ranged from 30 mg/kg bw per day in infants to 506 mg/kg bw per day in toddlers. The combined mean intakes of cellulose derived from their use as food additives seem to represent a very small proportion as compared to the dietary fiber coming from natural sources. To the best of our knowledge, no numerical ADI for CMC has been established up to now.

### Chemical contaminants in food

Xenobiotics are formed in industrial processes (*e.g.*, polycyclic aromatic hydrocarbons, polychlorinated biphenyls, or dioxins) or used in plant farming and animal husbandry (*e.g.*, pesticides) to boost production, reduce food waste, and ensure an adequate food supply (Figure 2(b)). Some of these compounds, such as dioxins, activate the Aryl hydrocarbon receptor (AhR) and Pregnane X receptor, both of which are key for intestinal homeostasis and xenobiotic/infection clearance. AhR activation leads to the release of antimicrobial peptides in the intestine that potentially modify microbial composition.^71^ This receptor has been linked to chronic intestinal inflammation.^72,73^ Overall, chemical contaminants in food are known to persist in the environment (air, soil, and water)^74^ and their bioaccumulation in the agri-food chain could represent a health risk leading to alterations in the human gut microbiome composition and function. Although studies with pesticide exposure report some degree of microbial disturbances, there are important limitations (*e.g.*, low statistical power associated to small sample size, lack of standardized models and analytical methodologies, limited consideration of confounding factors, lack of studies with focus on exposure science, limited knowledge of causal relationships and underlying mechanisms) that should be considered when interpreting the results of these studies and using their data for human health risk assessments.^75^ On the other hand, the intestinal microbiota can also metabolize xenobiotics, affecting toxicity, biological activity, and bioavailability. However, the microbial enzymes responsible for many of these transformations are still poorly understood.^18,20^

### Microplastics as emerging contaminants in food

Microplastics and other plastic-associated contaminants are recognized as emerging and ubiquitous contaminants with unknown health implications,^76,77^ despite the lack of current legislation to regulate them as food contaminants. Microplastics are widely distributed due to the overuse of plastics nowadays, and they are inadvertently ingested with our diet, partly because of their small size (<5 mm diameter). Microplastics exhibit a low degradation rate that facilitates their accumulation in various tissues and organs, such as liver, colon, lungs, or placenta.^78–80^ The oral route represents an important exposure pathway in humans.^81,82^ Seafood is one of the most prevalent sources of human dietary exposure to microplastics, like polystyrene, polyethylene, or polypropylene, due to the contamination of the marine environment. Food processing is also an additional potential source of contamination through the release of plastic particles from food contact materials, among other phenomena.^83^ Based on the metadata analysis of 59 publications^84^ it was estimated the amount of microplastics that humans may ingest, which can serve as a basis for future investigations and risk assessments. On average, humans could be ingesting 0.1–5 g of microplastics weekly. Moreover, a qualitative analysis identified over 10,000 chemicals in plastics.^85^ A more recent report from the Food Agriculture Organization (FAO) shows estimations of human dietary exposure to microplastics via the consumption of selected foods (*i.e*., mussels, clams, shrimp, and prawns (considered together), oysters, salt, honey, refined sugar, and tap water). The dietary exposure was estimated using the highest 95th percentile consumption for the different foods across a range of developed and developing countries and, following an overly conservative scenario, the highest reported microplastic concentration for each commodity.^86^ As an example, the exposure to microplastics in high consumers of mussels can be as high as 3,200 microplastics/day while in high consumers of clams would reach 1,701 microplastics/day. The FAO report also provides a comprehensive table with scientific studies describing the exposure to microplastic following the consumption of different foods. Furthermore, the exposure to microplastics seems to be especially relevant for infants where different intake sources have been identified, including baby feeding bottles,^87^ breast milk storage bags,^88^ baby teats,^89^ or breast milk itself.^90^ A provocative study has shown a higher concentration of fecal microplastics in inflammatory bowel disease (IBD) patients than in healthy individuals. Intriguingly, the concentration of microplastics positively correlated with the disease severity, suggesting these particles as potential triggers of IBD pathology. The authors also reported that patients with a higher abundance of fecal microplastic consumed more plastic-packaged products.^91^ Based on *in vitro* and *in vivo* studies, microplastics could alter microbiome composition and function, barrier dysfunction, as well as induce immune responses^92,93^ (Figure 3). Table 4 provides further information about the impact of certain microplastics in different biological processes, including immunity, metabolic pathways, and the consequent activation of gut–liver or gut–brain axes. However, further evidence is needed to elucidate whether microbial alterations are the cause or consequence of either microplastic consumption or host’s response to the particles.^98^ Moreover, microplastics could also act as carriers by transporting contaminants (*e.g*., heavy metals, and phthalate esters), antibiotics, or pathogens, which could directly or indirectly affect the gut microbiota and contribute to the accumulation of hazardous substances, change exposure pathways, and share detrimental mechanisms.^99–101^ Consequently, the co-exposure of microplastics together with other chemicals could generate additional risks that need to be further investigated.

**Table 4.** Gathered information linking selected substances in microplastics with changes/involvement of specific bacterial taxa, microbial metabolites and/or other end-products, and potential mechanism(s) influenced/mediated by the gut microbiome leading to adverse effects in the host.

Compound	Gut microbiota bacterial taxa associated to xenobiotic exposure	Microbial metabolites and pathway(s) on or mediated by the gut microbiome	Potential adverse effect(s) in the host	Refs
Diethyl-hexyl phthalate (DEHP)	***In** vivo* (humans): Early-life DEHP exposure led to a decrease in *Rothia* sp. and *Bifidobacterium longum* in humans	Animal studies have shown that DEHP can act as an adjuvant to aberrantly enhance antigen-specific humoral immunity through oral or inhalation routes at a relatively high dose. In asthma models, oral exposure to DEHP promoted immunoglobulin (Ig)E and IgG1 humoral immunity, at least in part by altering the function of T follicular helper cells, which play an important role in regulating B cell function. In terms of DEHP exposure and IgM response in humans, intravenous DEHP exposure could alter IgM responses to vaccinations in newborns. The establishment of bacterial communities and development of the immune system is highly interactive during the early years of life. Thus, intravenous DEHP-mediated alterations in IgM vaccine responses may, at least in part, be due to gut microbiota dysbiosis. Another possible reason is that DEHP and its metabolites may directly affect B cell responses during infancy	Associated with allergy and asthma development in children	^94–97^
Bisphenol A (BPA)	***In** vivo* (mice): Increase of *Proteobacteria* and decrease of *Akkermansia*	Expression levels of intestinal tight junction proteins (zonula occludens-1 and occludin) decreased drastically, leading to increased intestinal permeability and elevated levels of endotoxin. Furthermore, BPA up-regulated the expression of Toll-like receptor 4 (TLR4) and phosphorylation of nuclear factor-kappa B (NF-κB) in the liver and increased the production of inflammatory cytokines, including interleukin (IL)-1β, IL-18, IL-6 and tumor necrosis factor (TNF)-α	Hepatic steatosis in mice	
Polystyrene	***In** vivo* (mice): Reduction of *Lachnoclostridium* and *Lactobacillus*, and an increase of *Proteobacteria*, *Actinobacteria*, and *Desulfovibrio*	Mucus secretion is decreased and intestinal permeability is increased. The results of serum metabolomics suggested that certain metabolic pathways were enriched, such as ABC transporter pathways, aminoacyl-tRNA biosynthesis, biosynthesis of amino acids, and bile secretion. Besides, neurotransmitter metabolites were also altered	Anxiety behaviors in mice	
	***In******vivo*** (mice): The relative abundances of *Firmicutes* decreased and α-*Proteobacteria*, *Actinobacteria* increased	Gut mucin secretion is reduced. The levels of hepatic triglyceride (TG) and total cholesterol (TCH) decreased. The transcriptional status of hepatic PPARγ and genes involved in TG synthesis, such as *Gpat*, *Dgat1*, and *Dgat2*, in the epididymal fat were significantly down-regulated.	Hepatic lipid-metabolism disorder in mice	

## The challenge of establishing the causal relationship of the gut microbiome with human health

Discriminating causality from association between the human gut microbiome and health effects represents one of the most significant and long-standing challenges in the microbiome field. Approaches to understand causation such as Koch’s postulates and Bradford Hill’s criteria have been traditionally considered. However, they are more suitable for the “one-pathogen” paradigm than for the human gastrointestinal tract, which harbors one of the most complex, interrelated, and abundant microbial ecosystems.^102^ Hence, it is important to advance from the simplistic view of “one-compound, one-bacteria, one-disease” as it is not plausible that one specific food or component causes complex multi-factorial disorders by modifying individual bacterial species or strains. An example is the proposed mechanism linking the relationship between diet and IBD pathogenesis where various food components including fiber, red meat, fat, and food additives (*e.g*., emulsifiers) interact with our microbiome to either strengthen or weaken intestinal barrier function.^103^ Thus, gaining a better understanding of the multi-causal nature of diseases and their etiology is needed to identify the most effective and convenient targets for preventive interventions.

**Figure 4.**
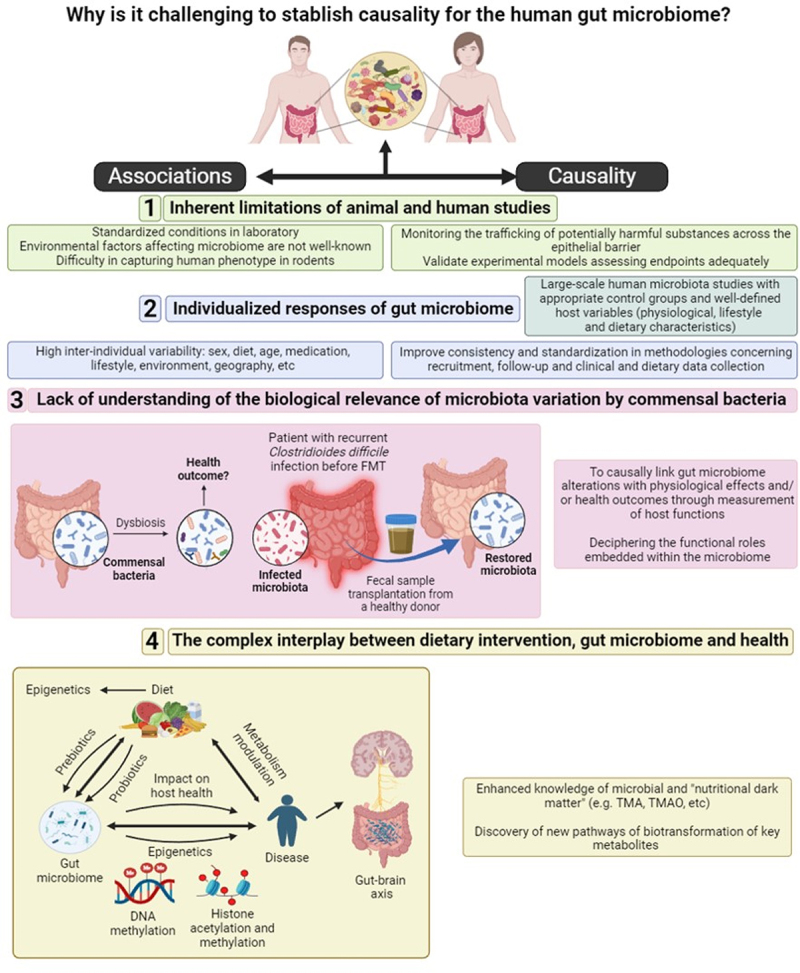
Main factors preventing the elucidation of causality in gut microbiome research studies and actions for their improvement. The causal role for the effects of dietary compounds mediated by the human gut microbiome is yet to be established as many of the outcomes of *in vitro*, *in vivo* animal and/or pre-clinical studies fail to recapitulate what is found in clinical studies. A shift from associations (left side) to causal relationships (right side) between a disrupted gut microbiome and adverse health effects in the host following interactions with specific dietary stressors is needed.

A number of relevant factors preventing the elucidation of causality in human gut microbiome research and corresponding actions to address this challenge are discussed below and summarized in Figure 4.

### The inherent limitations of animal and human studies in gut microbiome research

Although experiments in rodent models for studying diet–microbiota interactions are very informative due to the capacity of controlling many variables (genetics, diet, and microbiota), the translation of findings from animal models to humans needs to be carefully considered because of the difficulty in capturing human genetics, microbiome, and phenotypic diversity.^104^ Laboratory-animal studies are typically conducted with genetically homogeneous, in-bred animals maintained in standardized conditions, thus overlooking environmental and host factors that affect variability in the human gut microbiome.^105^

Observational studies are often used to establish associations between human exposure and health outcomes. Although epidemiological observations inform about real-life events in humans, they have many limitations, such as the lack of objective ways to assess dietary compliance (also impacting on RCTs) and the difficulty of implementing a true control group.^106,107^ Most studies typically compare healthy individuals with patients, failing to account for the impact of common environments and diets on the microbiota. This aspect could be better understood by including analysis of household controls and/or siblings.^108^ In addition, there are many confounding factors mostly related to diet variations (*e.g*., poor patient adherence to dietary regimes and large diversity of ingested food ingredients, diet recording approach, dietary changes influencing other host metabolism pathways in a microbiota-independent way) that preclude establishing a direct causal role between the intake of a specific dietary compound and a given health effect.^109^ Vujkovic-Cvijin et al.^110^ listed a series of host variables (physiological, lifestyle, and dietary characteristics) that should be controlled in human microbiota studies to match comparison groups. Considering these variables would increase robustness and reproducibility of microbiome signatures associated with human disease. Further translational studies (*e.g*., fecal microbiota transplantation (FMT) and interventions) are required, however, to ultimately prove causality.

Lastly, available animal and human model data sets are often small and normally use different assay methods which can be a limitation when comparing changes, particularly in risk assessment.

### Responses of the gut microbiome to diet are highly individual

The high inter-individual variability of gut microbiota and its evolving capacity hamper efforts in identifying a universally “normal” or “healthy” gut microbiota. Although humans present similar patterns of colonization after birth and core microbiota functions are highly preserved between individuals, the intestinal microbiota composition is different for each person. Consequently, the responses toward specific food components may vary depending on the microbiome of each person. A variety of factors, including diet, age, sex, medications, lifestyle, environment, geography, or ethnicity, shape the gut microbiome. The intra-host adaptation of the gut microbiota is thought to be shaped by age, immunity, and dietary habits in healthy individuals,^111^ determining the individual human gut microbiota’s ability to metabolize specific dietary components.

For example, the biotransformation pathway L-carnitine→γ-butyrobetaine→TMA/TMAO mediated by the human gut microbiota (Figure 1) is induced by omnivorous dietary patterns and chronic L-carnitine exposure, whilst vegans/vegetarians produce lower TMAO levels than omnivores.^112^ Similarly, the production of uremic *p*-cresol sulfate, which is generated by the gut bacterial metabolism of tyrosine (Figure 1), was also lower in vegetarians than in individuals consuming an unrestricted diet.^113^ The ability of certain animals, including humans, to convert cyclamate into cyclohexylamine (Figure 1) appears to depend upon a continuous intake of cyclamate.^114^ Several pathways involved in heterocyclic compound metabolism were enriched in saccharin-fed mice, suggesting that exposure to this NNS can be associated with an increase of saccharin-catabolizing bacteria.^115^ A similar finding was observed following consumption of the food additive xanthan gum, a complex polysaccharide used as a stabilizer and thickening agent. The authors showed that the ability to digest xanthan gum is common in human gut microbiomes from industrialized countries. This study revealed that the introduction of a new dietary component may drive changes in human microbiome with potential impacts on human health.^116^

Therefore, the notion of a “healthy” or “unhealthy” gut microbiota appears to be context-dependent. Determining an optimum combination of gut microbes and microbiome features considered universally healthy is not feasible in the short/medium term.^117^

### The biological relevance of gut microbiome variation triggered by commensal bacteria is unclear

A direct relationship between specific human gastrointestinal microbes and disease conditions has been firmly established only for a few diseases including peptic ulceration and gastric cancer linked to *Helicobacter pylori*, and antibiotic-associated diarrhea linked to *Clostridium difficile*.^104^ This is likely because both are single invasive pathogens of the stomach (*H. pylori*) and intestine (*C. difficile*), whose colonization and pathogenic mechanisms are well-understood.^118,119^ Indeed, FMT is an effective therapy for reversing microbial dysbiosis and treating recurrent or refractory *C. difficile* infection (CDI)^120^; also, an orally administered microbiota-based therapeutic product for preventing the recurrence of CDI in adults has been recently approved.^121^ Yet, the utility of FMT beyond the treatment of CDI is still limited and controversial,^122^ and further knowledge of the underlying ecological dynamics is required to understand its potential for other applications. FMT has been less successful in restoring the disrupted gut microbial ecosystem in other diseases which etiologies involve networks of bacterial species and metabolites and/or in diseases where the microbiome is not so severely disrupted (*e.g*., Crohn’s disease, metabolic syndrome) as in CDI. Recent studies suggest, for example, that other factors such as the similarity between donor and baseline recipient microbiota, as well as the sex and age, could be determinants for successful FMT trials in the amelioration of metabolic syndrome markers.^123^ The United States Food and Drug Administration (FDA) has developed a policy to help facilitate access to FMT for patients with CDI not responding to standard therapies.^124^ In this context, promising advances have been recently developed based on the use of engineered native bacteria (*e.g., Escherichia coli*) as chassis to functionally manipulate the gut microbiome of conventionally raised mice.^125^ Likewise, the relevance of the gut-lung axis through their respective microbiomes is increasing in respiratory allergies.^126^ A significant association between probiotics supplementation and reduced risk of respiratory allergy could not be found during a meta-analysis of data obtained from the RCT of probiotics usefulness for the protection against asthma incidences in infants.^127^ In addition, one must be very cautious with such treatments in preterm infants, because probiotics might predispose highly vulnerable populations to infection.^128^

Unlike the above examples, most gut microbiome studies have established associations between changes in commensal microorganisms and different pathologies. These changes, however, are frequently described by vaguely used terms like “dysbiosis”, “imbalance of microbiota”, or “reduced microbial diversity”, and do not always consider the causal links to physiological and/or health outcomes. Furthermore, the assumption that a decreased gut microbial diversity is generally associated with an unhealthy state has been challenged by several studies. For instance, a greater bacterial richness was found in feces from colorectal cancer patients compared to healthy individuals, partially due to the presence of oral cavity-associated species which are rarely found in the healthy gut.^129^

Since the mechanisms by which “dysbiosis” could cause disease are still under investigation and the significance of most microbiota fluctuations to disease remains speculative, the term dysbiosis alone does not indicate whether it is cause or consequence.^130,131^ Indeed, it is necessary to differentiate between harmless and harmful microbiome fluctuations understood by those that are dysfunctional, breaking the host-microbe symbiosis and adversely impacting human health. The term dysbiosis is also biased as it refers to deviations/differences from a “healthy baseline” which is not even close to being defined as previously mentioned. Therefore, the concept of dysbiosis has limited value in risk assessment unless supported by physiological and/or clinical outcomes and molecular mechanisms. The diversity and type of functions encoded by the microbiome may be more relevant than the alterations in the composition to define resilient or dysfunctional microbiome.^132^ Indeed, one of the ecological causes of gut dysbiosis seems to be an increased availability of host-derived respiratory electron acceptors. These are dominant drivers of microbial community composition by determining which redox reactions are available for microbial growth. Therefore, the underlying cause for alterations in the microbiota composition could be a change in the host environment.^133^ Consequently, the analysis of the metagenome and their products and how they modify host functions could provide more insights into the biological role of the microbiome than the current taxonomic analysis often performed.^134^ However, unraveling the functional roles encoded by the microbiome is much more expensive and complex than performing a taxonomic profiling.^135^

### The complex interplay between dietary intervention, gut microbiome, and health

Associations between gut microbes, gene microbial paths and functions, and the intake of specific nutrients, foods, and dietary patterns have been made by large-scale and high-quality metagenomic studies in well-phenotyped individuals.^136–138^ Even though, the studies have been mainly focused on prokaryotic microorganisms so far and results are inherently biased toward the most abundant ones.^139^ Furthermore, other microbes inhabiting the gut such as eukaryotic fungi and viruses have been overlooked until very recently.

Moreover, the bulk of our current understanding of how diet affects health is limited to ~150 key nutritional components, representing only a minute subset of the total pool of distinct and definable biomolecules present in foods, estimated to be ~27,000. This incomplete biochemical profiling of food components, defined as “nutritional dark matter,”^140^ adds uncertainty to the health implications of our diet and its interaction with the gut microbiome on human health.

Therefore, the generation of further knowledge on the relationship between the gut microbiome and its interactions with dietary and food composition should help to unravel their role in human health. This could lead to the elucidation of new microbiota-derived metabolites and bio-transformation pathways with biological impact. Likewise, in addition to the analysis of collective genes of microbes (metagenomics), their expression (metatranscriptomics) and possible epigenetic modifications (*e.g*., DNA methylation or histone modification) could play a key role in the pathogenesis of gastrointestinal^141–143^ and metabolic diseases.^144^ Interestingly, diet can influence the host epigenetic modifications through gut microbiome-derived metabolites.^145,146^

## Consideration of gut microbiome science in current food/feed safety assessments

Despite the complexity of the gut microbiome ecosystem and our limited understanding of causal mechanisms due to specific changes in gut microbiome components, the role of the gut microbiome in the safety assessment of certain food/feed compounds has been already considered, especially in those having antimicrobial effects. Thus, the impact of veterinary antimicrobial drug residues, including their metabolites, in animal-derived food products on the human gut microbiome is part of their safety assessment. This is based on a decision tree included in the internationally accepted Guideline VICH GL36 (R)^147^ which proposes two relevant microbiological endpoints: i) the disruption of the colonization barrier, and ii) the determination of the increase in the population(s) of resistant bacteria (*i.e*., antimicrobial bacteria), for exposure to residues of veterinary drugs.^148^ If appropriate, the estimation of a microbiological acceptable daily intake (mADI) is also described for chronic exposure since it is considered unlikely that a single exposure to residues of veterinary drugs would provide sufficient selective pressure to enable the emergence of a resistant bacterial population. Likewise, in the EFSA’s guidance on risk assessment of nanomaterials to be applied in the food and feed chain is envisaged the study of the effects on the gut microbiota composition exerted by certain insoluble/persistent nanomaterial, especially when having antimicrobial effects (e.g., some metals or metal oxides).^149^ Several steps based on validated *in vitro* gastrointestinal simulators inoculated with human gut microbiota, used as preliminary screening methods for nanomaterials to study microbial metabolism and effects on gut microbiota composition, followed by conventional animal models, leaving germ-free models as a final step are proposed in a tiered approach.

Another example is the Roxarsone (3-nitro-4-hydroxyphenylarsonic acid), which is a common organic arsenic-based additive traditionally used in chicken feed to improve the rate of weight gain, feed efficiency, and pigmentation, as well as control and treat bacterial and coccidial infections. The risk assessment carried out by the FDA showed the partial biotransformation of this compound into inorganic arsenic, which is much more toxic than the organic form, through *in vitro* fecal fermentation studies.^150^ The metabolization of this type of arsenic-based feed additives by the poultry gut microbiome may contribute to the human dietary exposures to arsenic, including inorganic arsenic, and in the environmental distribution of arsenic in manure.^151^ As a consequence, the FDA has since withdrawn approval for any organo-arsenic animal drugs to be used in the United States.^152^ Moreover, for the food safety assessment, there is an increase in the microbiome and omics-based approaches for the identification of bacterial contaminants.^153,154^ Novel approaches, such as whole-genome sequencing, metagenomics, and metabolomics, are used to determine an outbreak, track the origin of contamination, virulence profiling, and monitor the antimicrobial resistance profile of bacterial pathogens in humans.^155,156^

## Concluding remarks and future perspectives

The detrimental impact of some dietary xenobiotics, such as certain food additives and chemical contaminants on human health, is partly mediated by the gut microbiome and its ability to modify and respond to these xenobiotics. In addition, the variation in the microbiome across individuals may explain why people show differential responses to these compounds. However, these potentially harmful xenobiotics are clinically under-studied, and most of the collected research has been conducted either *in vitro, ex vivo*, or in rodent models.^157^ This scenario, together with the multiple factors involved in gut microbiome dynamics (high inter-individual variability, diet, ethnicity, *etc*.) and characterization (lack of consistency and standardization in microbiome analysis, sampling, or recruitment), often hampers the elucidation of how dietary modulators and the gut microbiome could interact leading to adverse health outcomes (Figure 4).

**Figure 5.**
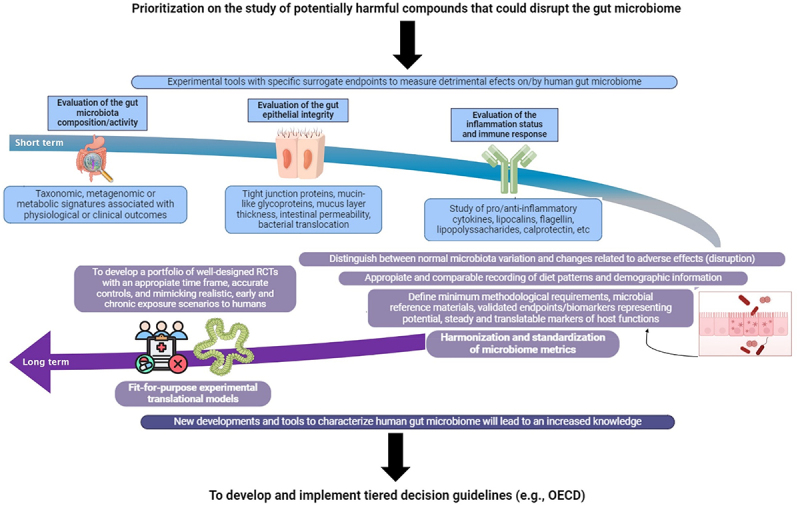
Starting proposed roadmap for the future incorporation of gut microbiome research in food safety risk assessment. This roadmap provides recommendations for developing a harmonized approach to use gut microbiome research studies in the regulatory decision-making process. First, a battery of validated experimental tools, assays, and endpoints could be incorporated in the short/medium term if international consensus is achieved. Second, there is a need for new developments and tools to address the identified gaps in the medium/long term. If these actions are successfully accomplished, the assembly and appraisal of the gathered information will be useful for the future development of standardized guidelines for the risk assessment of some specific stressors of the human gut microbiome.

The safety or risk assessment of specific food components of concern should consider the effects on, or mediated by, the gut microbiome in the near future. These studies together with the well-established toxicity and mutagenic trials, could help to fully understand potential health hazards. A roadmap is needed to incorporate microbiome science in food risk assessment (Figure 5). This roadmap should encompass: a) a prioritization strategy for dietary compounds that are increasingly present in modern diets and that have been identified as potentially harmful to the human gut microbiome and health; b) the development and incorporation into the food risk assessment in the short/medium term of a series of experimental tools with specific microbiome-related and host health endpoints to measure gut microbiome perturbations (Figure 5). These studies, whose outcome might further support the safety assessment of certain food contaminants and/or additives, could include the evaluation of the:

- Gut microbiota composition and metabolism when causally linked to physiological and/or clinical outcomes and supported by molecular mechanisms.
- Gut epithelial integrity and permeability.
- Inflammation status and immune response.

There is also the need for the development of new approaches and tools to structurally and functionally characterize the human gut microbiome, providing enhanced knowledge to guide future research into the relationship between dietary components and the microbiome in risk assessment (Figure 5). We suggest focusing on the following key elements:

- Define the type and levels of functional microbiota modulation that are biologically relevant.
- Identify indicators demonstrating the disruption of the gut microbiome (*i.e*., minimum methodological requirements, microbial reference materials, and validated endpoints/biomarkers/metabolites that could represent potential, steady, and translatable markers of the host functions).
- Identify relevant translational research methods to connect *in vitro* and *in vivo* animal outcomes to the human context.
- Develop a portfolio of well-designed RCTs with appropriate time frames and time-series data, accurate controls, and mimicking realistic, early, and chronic exposure scenarios to humans.
- Accelerate harmonization and standardization of microbiome methodologies and metrics to obtain high-quality comparable evidence. This includes consistent recording of diet patterns and demographic information.

These key actions should drive future long-term research goals to enhance our knowledge into the adverse outcomes derived from the complex triad involving certain dietary modulators, gut microbiome, and host health. More specifically, it is pivotal to gain, among other aspects, a deeper understanding of: i) causal mechanistic pathways (*i.e*., molecular, cellular, and tissue/organ effects including their potential role in regulating intestinal mucosal barrier integrity) and clinical aspects (*i.e*., adverse effects involved in the pathogenesis of certain gastrointestinal-related disorders) associated with the interaction of dietary xenobiotics with the human gut microbiome; ii) common key chemical features and/or functionality of potentially harmful substances that could influence adverse outcomes; iii) underlying factors and determinants driving inter-individual variability (*e.g.*, genetic predisposition in autoimmune diseases, evolution of the gut microbiota in response to the dietary habits, environmental and life-style factors); iv) the biological meaning of a structural or functional disrupted gut microbiome (*i.e*., to elucidate the nature and magnitude of change in the microbiome that might result in adverse health effects and discriminate these changes from normal fluctuations); v) metagenome-assembled genomes from food and food-related environment samples harboring antimicrobial resistance genes (ARGs); vi) the role of antimicrobial and non-antimicrobial compounds in the distribution and transmission of ARGs; and/or vii) the role of disrupted gut microbiome in the horizontal gene transfer of ARGs or pathogenicity genes from microorganism-based products to gut microbiota.

The achievement of new basic research findings and technological developments, as described above, will lead to increase the robustness, avoid inconsistencies, and lack of reproducibility of gut microbiome research data. These actions will facilitate the future development and implementation of standardized tiered decision guidelines (*e.g.*, such as those of the Organisation for Economic Co-operation and Development (OECD)) (Figure 5). Ultimately, the prescription of specific data requirements included in the regulation could be incorporated on an *ad hoc* basis into existing risk assessment frameworks for some specific stressors of the human gut microbiome.

## Note

1. https://open.efsa.europa.eu/questions?search=saccharine.
